# β-Catenin Phosphorylated at Serine 45 Is Spatially Uncoupled from β-Catenin Phosphorylated in the GSK3 Domain: Implications for Signaling

**DOI:** 10.1371/journal.pone.0010184

**Published:** 2010-04-16

**Authors:** Meghan T. Maher, Rigen Mo, Annette S. Flozak, Ofra N. Peled, Cara J. Gottardi

**Affiliations:** 1 Department of Medicine, Northwestern University Feinberg School of Medicine, Chicago, Illinois, United States of America; 2 Integrated Graduate Program in the Life Sciences, Northwestern University Feinberg School of Medicine, Chicago, Illinois, United States of America; 3 Department of Biological Sciences, National Louis University, Chicago, Illinois, United States of America; University of Washington, United States of America

## Abstract

*C. elegans* and *Drosophila* generate distinct signaling and adhesive forms of β-catenin at the level of gene expression. Whether vertebrates, which rely on a single β-catenin gene, generate unique adhesive and signaling forms at the level of protein modification remains unresolved. We show that β-catenin unphosphorylated at serine 37 (S37) and threonine 41 (T41), commonly referred to as transcriptionally Active β-Catenin (ABC), is a minor nuclear-enriched monomeric form of β-catenin in SW480 cells, which express low levels of E-cadherin. Despite earlier indications, the superior signaling activity of ABC is not due to reduced cadherin binding, as ABC is readily incorporated into cadherin contacts in E-cadherin-restored cells. β-catenin phosphorylated at serine 45 (S45) or threonine 41 (T41) (T41/S45) or along the GSK3 regulatory cassette S33, S37 or T41 (S33/37/T41), however, is largely unable to associate with cadherins. β-catenin phosphorylated at T41/S45 and unphosphorylated at S37 and T41 is predominantly nuclear, while β-catenin phosphorylated at S33/37/T41 is mostly cytoplasmic, suggesting that β-catenin hypophosphorylated at S37 and T41 may be more active in transcription due to its enhanced nuclear accumulation. Evidence that phosphorylation at T41/S45 can be spatially separated from phosphorylations at S33/37/T41 suggests that these phosphorylations may not always be coupled, raising the possibility that phosphorylation at S45 serves a distinct nuclear function.

## Introduction

β-catenin is a prototypic example of a dual-function adhesion signaling protein. At the cell surface, β-catenin binds the cytoplasmic domain of cadherin-type adhesion receptors which, together with the actin-binding protein, α-catenin, allows cells to link their cytoskeletal networks through robust intercellular adhering junctions [1], [2], [3], [4]. In the cytoplasm and nucleus, a cadherin-independent pool of β-catenin transduces extracellular Wnt signals by interacting with TCF-type transcription factors to activate target genes that control cellular differentiation. In *C. elegans*, dedicated signaling and adhesive forms of β-catenin are specified at the level of three distinct genes [5]: HMP-2 interacts exclusively with the cadherin gene product, HMR-1, while BAR-1 and WRM-1 transduce Wnt signals through the TCF homolog, POP-1. Moreover, *Drosophila* make a neural splice form of β-catenin, which lacks the C-terminus and is used exclusively for neural cell adhesion [6]. Since vertebrates only rely on a single β-catenin gene, it has been speculated that vertebrates generate distinct signaling and adhesive forms of β-catenin through post-translational modification.

The best-known modifications of β-catenin are a series of phosphorylations that continually promote degradation of the cadherin-free pool of β-catenin. Specifically, CK1α phosphorylates β-catenin at S45, which primes this N-terminal region for subsequent phosphorylations by GSK3 at T41, S37 and S33 [7]. These latter two phosphorylations are recognized by the E3-ligase component, β-TrCP, for ultimate ubiquitylation and destruction by the proteosome [8], [9]. The scaffold protein, Axin, enhances the efficiency and tight coupling of these N-terminal phosphorylations due to its ability to bind β-catenin and both CK1α and GSK3 kinases [10]. The tumor suppressor protein, APC, binds β-catenin and shields these phosphorylations from the protein phosphatase PP2A, thus favoring their recognition by β-TrCP [11]. Loss-of-function mutations in APC or Axin essentially mimic Wnt activation by preventing GSK3β-mediated phosphorylation of β-catenin, which allows β-catenin to accumulate to high levels in both cytoplasmic and nuclear compartments [12]. While the accumulation of β-catenin is a clear hallmark of Wnt signaling, levels alone are *insufficient* to explain β-catenin signaling activity, as β-catenin that remains unphosphorylated at GSK residues 33, 37 and 41 is intrinsically more active than β-catenin that can be phosphorylated at these residues [13], [14]. A molecular explanation for why N-terminally unphosphorylated β-catenin is more transcriptionally active has remained elusive. Other recent studies indicate that β-catenin signaling activity can be enhanced by phosphorylations at Ser552 [15], Ser675 [16], [17] and Ser191 [18], [19], but whether these modifications serve to generate a dedicated signaling form of β-catenin remains unexamined.

The SW480 colon carcinoma cell line expresses low levels of E-cadherin and harbors a mutation in the tumor suppressor protein APC, resulting in an inability to properly degrade N-terminally phosphorylated β-catenin [20], [21]. As a result, these cells have elevated nuclear and cytoplasmic β-catenin. More important, since the Axin-scaffold complex is fully operational in these cells, these normally short-lived N-terminal phospho-forms of β-catenin accumulate to a level that facilitates their detection by phospho-specific antibodies. Thus APC mutant SW480 cells allow us to capture what is normally a transient intermediate step in the phospho-destruction of β-catenin [22]. Given evidence for distinct signaling and adhesive forms of β-catenin in *C. elegans*, and *Drosophila* [5], [6], we sought to characterize molecular properties of the nuclear and cadherin-binding form of β-catenin using recently developed antibodies that recognize distinct phospho-forms of β-catenin.

## Results

### ABC is a minor subpopulation of total β-catenin

**Figure 1:**
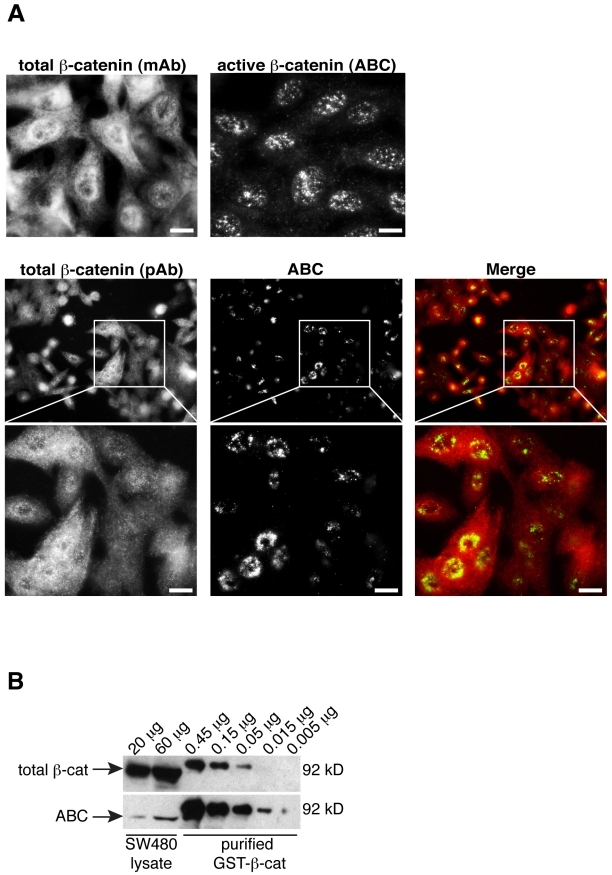
N-terminally unphoshorylated β-catenin is a minor, nuclear form of β-catenin. A) Immunofluorescence of SW480 cells for total β-catenin or ABC (active β-catenin). Both monoclonal (mAb) and polyclonal (pAb) antibodies against total β-catenin produce a pan-cellular staining, while ABC predominantly stains nuclei. B) SW480 lysate (20 or 60 µg) was compared to known quantities of purified GST-β-catenin to estimate the relative abundance of total β-catenin to ABC. ABC is approximately 100 times less abundant than total β-catenin. Bars, 10 µm.

Using a monoclonal antibody that recognizes the transcriptionally **A**ctive form of **β-C**atenin (ABC, specifically unphosphorylated at serines 37 and threonine 41; [14], [23]), we observe a predominantly nuclear staining pattern in SW480 cells lacking E-cadherin (Fig. 1A). Since an antibody that recognizes a C-terminal epitope of β-catenin stains both cytoplasmic and nuclear compartments (total β-catenin, Fig. 1A), it appears that ABC might be a distinct subpopulation of total β-catenin. Indeed, ABC is approximately 100 times less abundant than the total pool of β-catenin in SW480 cells, using a standard curve of β-catenin purified from bacteria and a widely used C-terminal antibody to β-catenin (Fig. 1B). ABC is similarly less abundant in primary epithelial cells that exhibit Wnt/β-catenin signaling in freshly isolated cultures [24]. These data suggest that the signaling form of β-catenin is a minor, nuclear-enriched population of the total *cytosolic pool* of β-catenin.

### Cytosolic ABC is primarily monomeric

**Figure 2:**
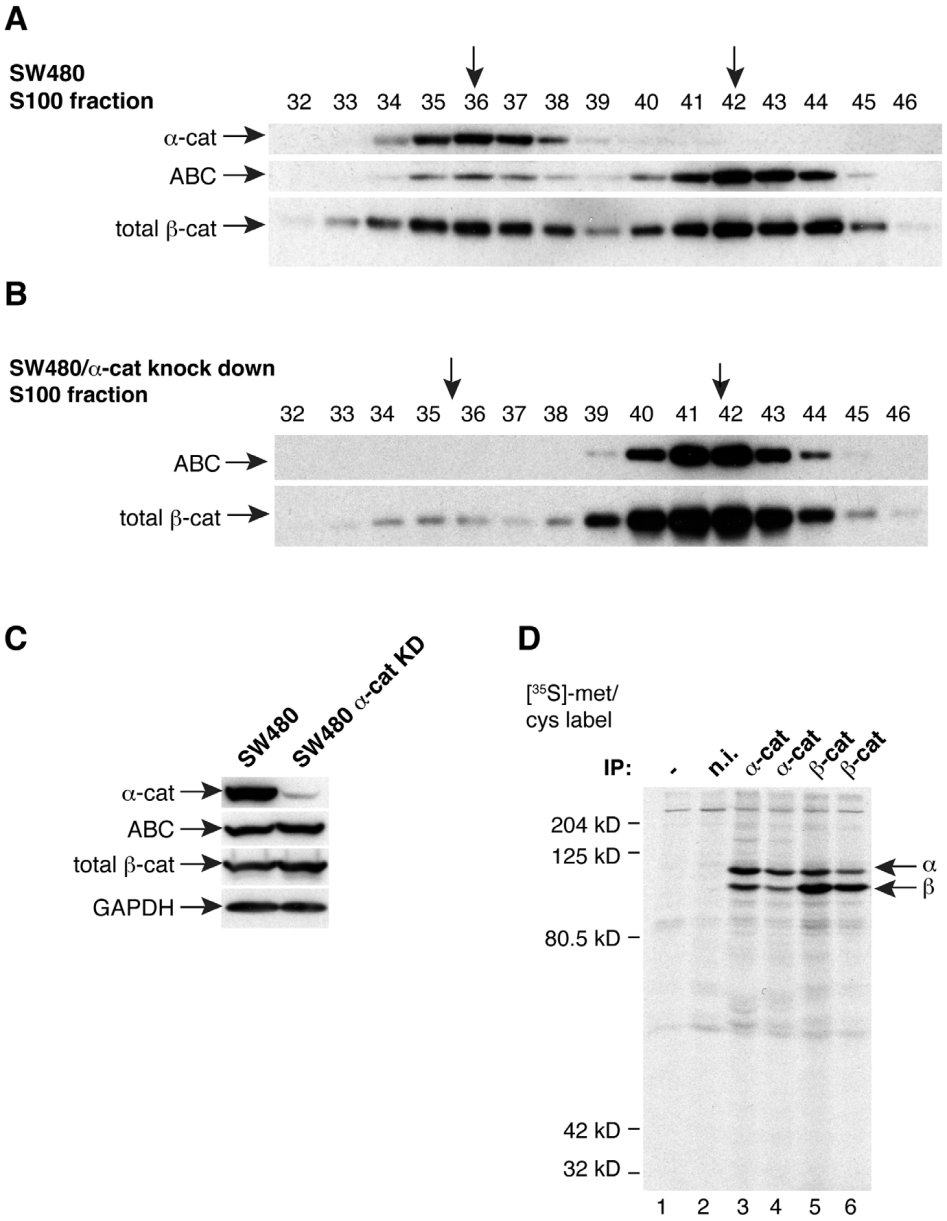
Cytosolic N-terminally unphosphorylated β-catenin is primarily monomeric. A detergent-free cytosolic fraction from SW480 control (A) or α-catenin shRNA knock-down (B) cells was subjected to gel filtration chromatography and immunoblot analysis. A) ABC sizes as a monomer (compared to calibration standards (not shown) and purified β-catenin (Fig. S1); peak fraction #42), while total β-catenin sizes evenly between monomer- and β-catenin/α-catenin dimer fractions (peak fraction #36). Peak fractions are marked with arrows. B–D) β-catenin dimer fraction is due to association with α-catenin. B) Size fractionation of cytosol from SW480 cells depleted of α-catenin by shRNA. C) Immunoblot of SW480 control and α-catenin knock down lysates. D) [^35^S]-methionine/cysteine-labeling of SW480 cells and immunoprecipitation of α-catenin and β-catenin (1∶100, lanes 3 and 5; 1∶300, lanes 4 and 6). Autoradiogram reveals the major binding partner of β-catenin in this cell type is α-catenin. No antibody (lane 1) or non-immune control (lane 2) are also shown.

Two distinct pools of β-catenin accumulate in the cytosol during Wnt signaling: β-catenin monomers and α-catenin/β-catenin heterodimers [25]. Given indications that β-catenin monomers may comprise the signaling form of β-catenin [25], [26], and that α-catenin binding to β-catenin can attenuate signaling [27], [28], we sought to determine the size-fractionation characteristics of ABC in SW480 cells. Gel filtration chromatography of SW480 cytosol reveals that ABC largely runs as a single peak fraction (Fig. 2A; #42; albumin at fraction #47, not shown), while total β-catenin runs as two peak fractions (Fig. 2A; #36 and 42). Since the chromatographic profile of purified myc-tagged β-catenin is similar to ABC (Fig. S1), these data suggest that cytosolic ABC is mostly a monomeric form of β-catenin. The larger molecular size peak fraction of β-catenin reflects β-catenin/α-catenin dimers, as this peak perfectly cofractionates with α-catenin (Fig. 2A), shRNA knock-down of α-catenin eliminates this size fraction of β-catenin (Fig. 2B & C), and α-catenin is the major binding partner of β-catenin in these cells (Fig. 2D). Altogether, these data indicate that cytosolic β-catenin which remains unphosphorylated at S37 and T41 is less likely to be in a complex with α-catenin.

### ABC is selectively recruited to cell-cell contacts

**Figure 3:**
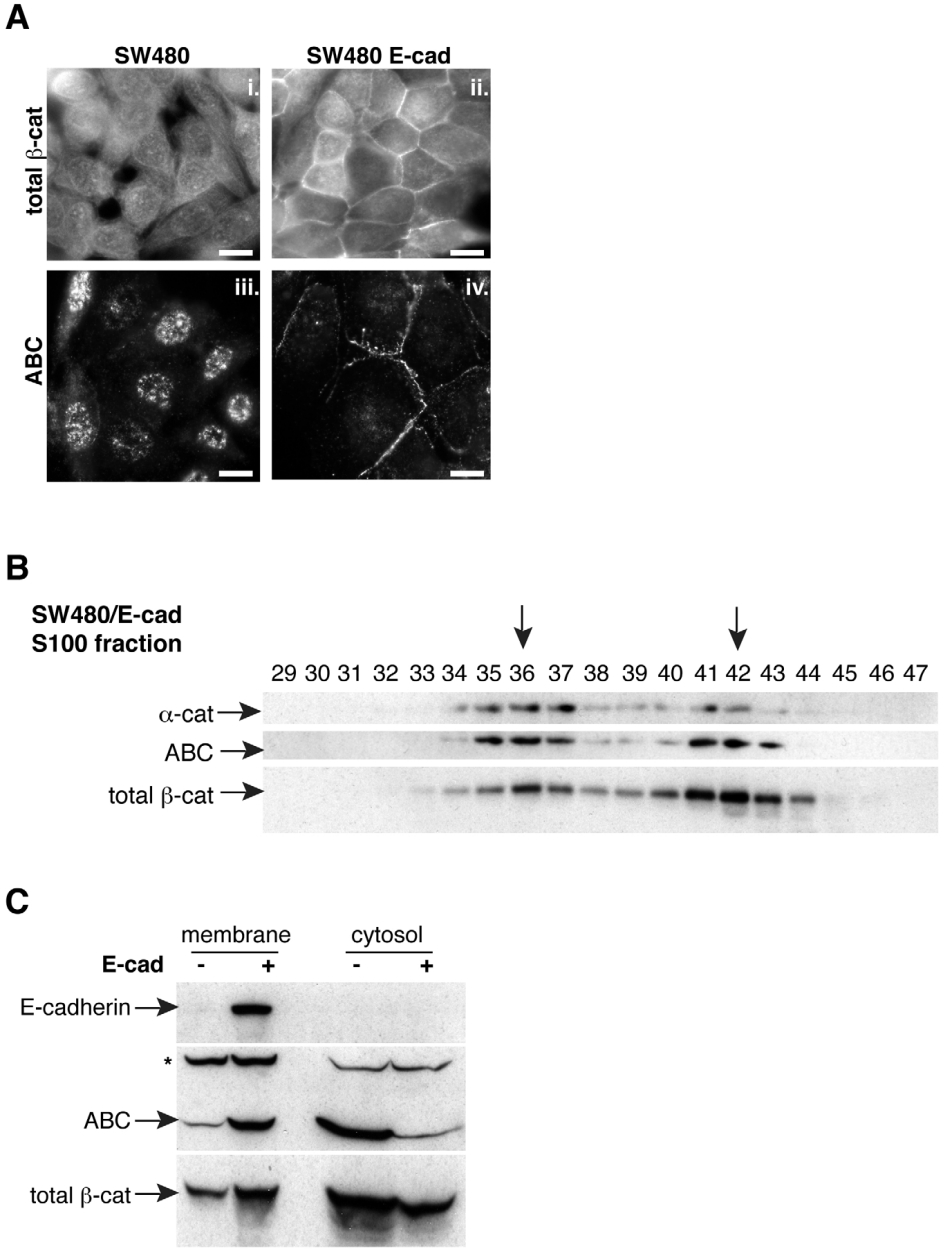
N-terminally unphosphorylated β-catenin appears highly sensitive to cadherin expression. A) Control (i. and iii.) and E-cadherin-restored SW480 cells (ii. and iv.) were stained with antibodies against total β-catenin or ABC. Note that ABC appears selectively recruited to sites of cell-cell contact upon cadherin expression relative to the total pool of β-catenin. B) A detergent-free cytosolic fraction from SW480/E-cadherin cells was subjected to gel filtration chromatography. Peak fractions are marked with arrows. The presence of E-cadherin reduces the abundance of monomeric ABC relative to control cells (compare to Fig. 2A). C) Detergent-free preparations of membrane and cytosolic fractions isolated from control and E-cadherin-restored SW480 cells. Note that E-cadherin appears to selectively recruit ABC to the membrane, leaving a portion of total β-catenin in the cytosol. A non-specific band (*) recognized by ABC [47] serves as a loading control. Bars, 10 µm.

The prominent nuclear pattern of ABC observed in Fig. 1 was also previously observed in Wnt-activated HEK293T cells [14] and within intestinal crypt cells [23], suggesting that ABC might be used exclusively in nuclear signaling rather than adhesion. However, ABC can be recruited to sites of cell-cell contact in E-cadherin-restored SW480 cells (Fig. 3A). Remarkably, the nuclear pool of ABC appears completely diminished compared with an antibody that recognizes “all” forms of β-catenin (i.e., “total β-catenin”; Fig. 3A). Restoration of E-cadherin also changes the chromatographic profile of ABC, reducing the relative fraction of monomeric ABC (Fig. 3B), and causes an apparent shift in ABC from cytosol to a crude membrane fraction (Fig. 3C), suggesting that ABC is selectively targeted by E-cadherin. Membrane recruitment of ABC is largely due to direct binding to the cadherin, since SW480 cells expressing an E-cadherin lacking the β-catenin binding domain (E-cadΔ35) show little ABC membrane staining (not shown). Thus contrary to initial indications from immuno-labeling studies [14], [23], β-catenin that remains unphosphorylated at S37 and T41 (ABC) is not refractory to cadherin-binding, but rather appears to be selectively targeted by cadherins.

### N-terminally phosphorylated β-catenin is mostly not associated with cadherins

**Figure 4:**
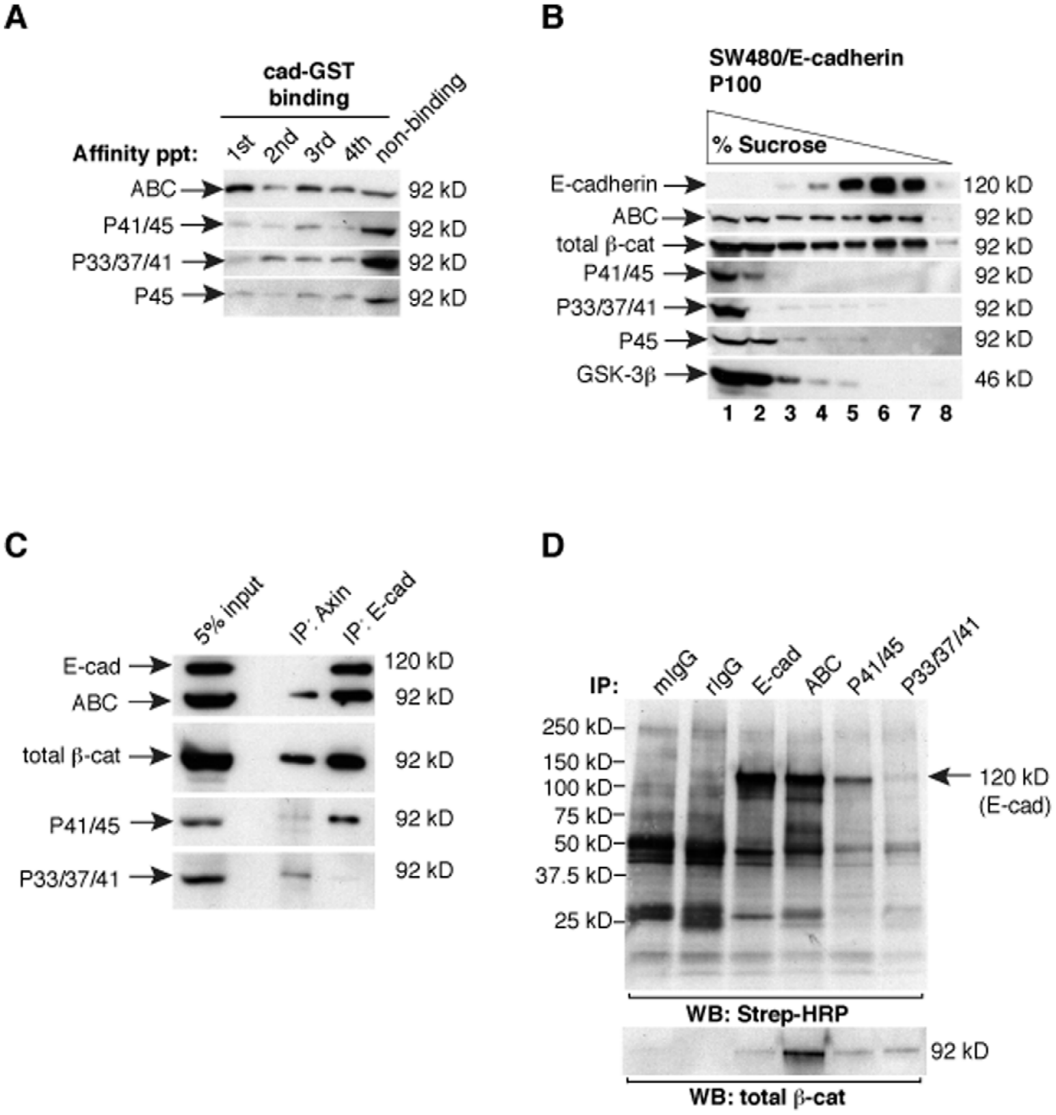
N-terminally phosphorylated β-catenin is largely not associated with E-cadherin. A) SW480 cell lysates were sequentially incubated with GST-cadherin cytoplasmic domain coupled-glutathione sepharose beads. Non-binding lane reflects 5% of the total unbound fraction. Note that while ABC can be affinity precipitated by GST-cadherin, β-catenin phosphorylated at S45, T41/S45, and S33/37/T41 bind to a lesser extent. B) Sucrose gradient density centrifugation of the detergent-free membrane preparation from SW480/E-cadherin cells reveals that N-terminally phosphorylated β-catenin does not appreciably co-fractionate (i.e., float) with cadherins. C) Immunoprecipitation of Axin or E-cadherin from SW480/E-cadherin lysates reveals that β-catenin phosphorylated at S33/37/T41 does not associate with E-cadherin. D) Cell surface biotinylation of SW480/E-cadherin cells followed by immunoprecipitation with the indicated antibodies and detection by streptavidin-HRP reveals that ABC coimmunoprecipitates with a cell surface protein the same size as E-cadherin, while β-catenin phosphorylated at S33/37/T41 does not. Western blot analysis for total β-catenin confirms that the same amount of β-catenin was immunopreciptated with antibodies against T41/S45 and S33/37/T41. Mouse IgG (mIgG) and rabbit IgG (rIgG) controls are shown, as well as a positive control for E-cadherin (IP: E-cad).

Evidence that ABC is a minor form of total cellular β-catenin (Fig. 1B), which can be selectively sequestered by cadherins (Fig. 3), implies that most of the β-catenin in SW480 cells is modified at the S37/T41 epitope and exists in a form that cannot be sequestered by cadherins. Since the N-terminal phosphorylation of β-catenin is thought to occur exclusively within the Axin-scaffold complex [7], [29], where APC binds β-catenin and shields these phosphorylations from the protein phosphatase PP2A [11], we reasoned that N-terminally phosphorylated β-catenin remains captured by APC and Axin, and is thus unable to bind E-cadherin. To test this hypothesis, we subjected SW480 whole cell lysate to sequential affinity precipitations with purified E-cadherin cytoplasmic domain (GST-E-cad cyto). We find that ABC associates with GST-E-cad cyto more readily than the phospho-forms of β-catenin, which largely remain in the non-binding fraction (Figs. 4A and S2). Consistent with this *in vitro* binding assay, N-terminal phospho-forms of β-catenin do not co-fractionate with E-cadherin compared to ABC by sucrose density “flotation” analysis (Fig. 4B). Instead, phospho-β-catenins co-sediment with degradation complex components in dense fractions as shown previously [22]. In agreement with these fractionation data, ABC, but not β-catenin phosphorylated at S33/37/T41, coimmunoprecipitates with E-cadherin (Fig. 4C). Curiously, some β-catenin phosphorylated at T41/S45 co-immunoprecipitates with E-cadherin (Fig. 4C). Moreover, while E-cadherin appears to be the main surface protein that associates with phospho-T41/S45 using the membrane-impermeant biotinylation method (Fig. 4D), this association appears to be minor, since most of the phospho-T41/S45 β-catenin present in a crude membrane fraction fails to float with E-cadherin and ABC (Fig. 4B). Altogether, these data indicate that *most* of the cadherin-bound β-catenin is unphosphorylated at both CK1 and GSK sites. β-catenin phosphorylated at GSK sites S33/37/T41 cannot interact with cadherins, likely due to its association with phospho-destruction complex components Axin and/or APC. Evidence that a small proportion of β-catenin phosphorylated at T41/S45, but not at S33/37/T41, can associate with the cadherin by co-immunoprecipitation analysis may be at odds with the prevailing model that β-catenin N-terminal phosphorylations occur exclusively within the Axin-scaffold complex.

### β-catenin phosphorylated at T41/S45 is spatially uncoupled from β-catenin phosphorylated at S33/37/T41

**Figure 5:**
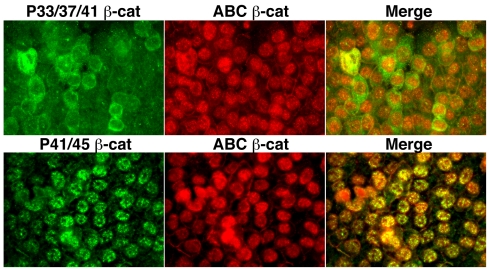
β-catenin phosphorylated at T41/S45 is spatially uncoupled from β-catenin phosphorylated at S33/37/T41. SW480 cells were co-stained with antibodies against β-catenin phosphorylated at S33/37/T41 or T41/S45 and ABC. Merged images reveal that phospho-S33/37/T41 appears excluded from the nucleus, while phospho-T41/S45 is largely nuclear, as is ABC. Bars, 10 µm.

The predominant nuclear localization of β-catenin unphosphorylated at S37 and T41 (ABC), compared to the even distribution of total β-catenin throughout both cytoplasmic and nuclear compartments (Fig. 1A), suggested that β-catenin phosphorylated at S33/37/T41 may be largely cytoplasmic. As predicted, antibodies against phospho-S33/37/T41 β-catenin recognize the cytoplasm to a greater extent than the nucleus (Fig. 5) raising the possibility that phosphorylation by GSK3 may impact β-catenin nuclear/cytosplasmic distributions. Intriguingly, β-catenin phosphorylated at T41/S45 does not exhibit the same distribution as β-catenin phosphorylated at S33/37/T41, and more strongly co-localizes with ABC in the nucleus (Fig. 5). Evidence that phospho-β-catenin is the major antigen recognized by these antibodies in SW480 cells was previously demonstrated [22] and is also shown in Fig. S3. Together, these data suggest that the nuclear form of β-catenin may be largely phosphorylated at S45 and unphosphorylated at S33, S37 and T41. Unfortunately, an antibody that exclusively recognizes β-catenin phosphorylated at S45 appears unsuitable for immunofluorescence analysis, but detects β-catenin in a nuclear-enriched fraction (not shown). Nonetheless, evidence that β-catenin phosphorylated at S33/37/T41 incompletely overlaps with β-catenin phosphorylated at T41/S45 raises the possibility that phosphorylation of β-catenin at the CK1α site may be spatially uncoupled from phosphorylations within the GSK-3β cassette.

### Molecular characterization of β-catenin phosphorylated at S552 and S675

**Figure 6:**
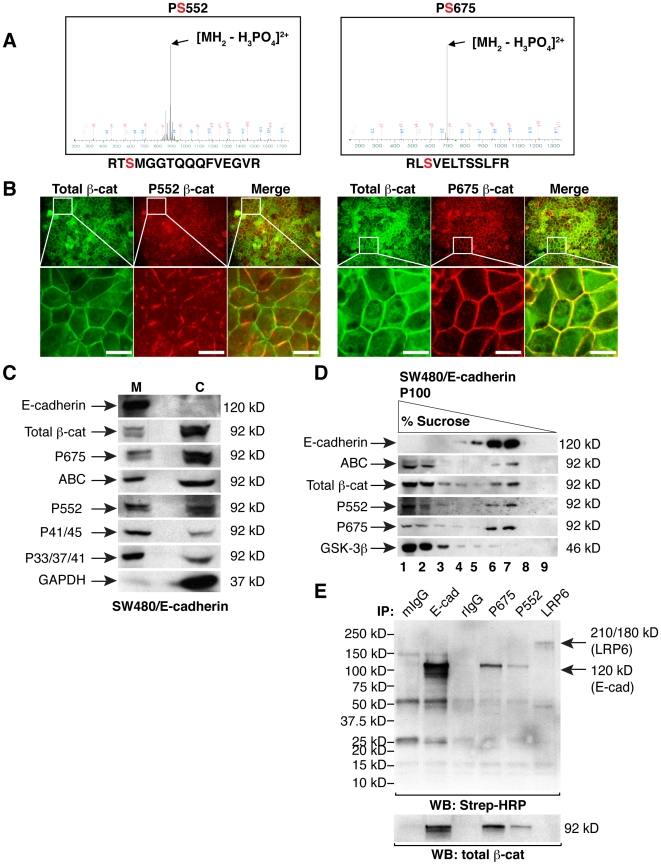
β-catenin phosphorylated at S552 or S675 localizes to cell contacts and associates with E-cadherin. A) Cadherin-free β-catenin was isolated from an SW480 lysate by affinity precipitation with GST-ICAT, as previously described [48]. LC-MS/MS analysis identified S552 and S675 as two phosphorylation sites in β-catenin. Peptide abundance is plotted as a function of mass/charge (m/z). Identified phospho-sites are shown in red. B) Immunofluorescence of SW480 cells with antibodies to total β-catenin and phospho-S552 or -S675 reveals that phospho-S552 appears punctate, while phospho-S675 and total β-catenin localize uniformly to sites of cell-cell contact. C) Detergent-free lysis of membrane and cytosolic fractions from SW480/E-cad cells. D) Sucrose gradient density centrifugation of the detergent-free membrane preparation from SW480/E-cadherin cells. Note that phospho-S552 or -S675 float with cadherins. E) Cell surface biotinylation of SW480/E-cadherin cells followed by immunoprecipitation with the indicated antibodies and detection with streptavidin-HRP reveals that phospho-S552 and -S675 coimmunoprecipitate with a cell surface protein the same size as E-cadherin. Western blot for total β-catenin demonstrates immunoprecipitation efficiency. Mouse IgG (mIgG) and rabbit IgG (rIgG) controls are shown, as well as a positive control for E-cadherin (IP: E-cad). An LRP6 immunoprecipitation was also performed and neither phospho-S552, -S675 nor N-terminal phospho-forms of β-catenin coimmunoprecipitate with a surface protein of this size. Bars, 10 µm.

Phosphorylation of β-catenin at S552 or S675 (corresponding to the C-terminal armadillo repeat region of β-catenin) has been each associated with increased signaling activity [15], [16], [17], but precise mechanisms for the enhanced nuclear signaling remain unclear. Using Liquid Chromatography tandem Mass Spectrometry (LC-MS/MS), we find that a cadherin-free pool of β-catenin precipitated from SW480 cell lysates can be phosphorylated at serines 552 and 675 (Fig. 6A). Nonetheless, β-catenin phosphorylated at each of these sites can localize readily to cell-cell contacts (Fig. 6B), co-fractionate with cadherins by sucrose equilibrium density flotation analysis (Fig. 6 C & D) and associate with a cell surface protein that perfectly co-migrates with E-cadherin (Fig. 6E). Thus, although β-catenin phosphorylated at S552 or S675 has been associated with increased transcriptional activity [15], [16], [17], this is not apparently due to an inability to associate with cadherins. Of interest, β-catenin phosphorylated at S552 or S675 reveal very different cell contact staining patterns. While antibodies that recognize phospho-S675 uniformly label cell contacts, antibodies to phospho-S552 reveal a punctate pattern that decorates only a subdomain of the cell contact (Fig. 6B). The localization of these phospho-β-catenins at cell contacts raises the possibility that these phosphorylations may also contribute to membrane proximal signaling events.

## Discussion

While the accumulation of cytosolic β-catenin is an established hallmark of Wnt signaling, it is also appreciated that β-catenin transcriptional activity can be observed in the absence of robust nuclear accumulation. In addition, small changes in β-catenin protein levels can correspond to dramatic tissue phenotypes known to rely on β-catenin signaling [30], [31]. These observations can be explained, at least in part, by evidence that β-catenin protein levels alone are insufficient to explain its signaling activity (13). Indeed, β-catenin that remains unphosphorylated at GSK residues 33, 37 and 41 is intrinsically more active than β-catenin that can be phosphorylated at these residues [13], [14]. Why an N-terminally unphosphorylated β-catenin is more transcriptionally active, however, has remained elusive. The present study offers a molecular explanation for these observations. We show that the cadherin-free form of β-catenin unphosphorylated at S37 and T41, typically referred to as transcriptionally active β-catenin (ABC), is a minor nuclear-enriched monomeric form of β-catenin.

The low abundance of ABC either in APC mutant SW480 cells (1–10% of the total pool of β-catenin, Fig. 1), or Wnt-activated primary epithelial cells [24], may explain why this form of β-catenin can be difficult to detect in the nuclei of cells activated by Wnt. These data further imply that the inhibitory phosphorylation of β-catenin at GSK3 residues S33/37/41 is robust, even in cells that exhibit constitutive activation of β-catenin signaling through loss of APC function.

Our data also show that β-catenin unphosphorylated at S37 and T41 is almost exclusively nuclear, while β-catenin phosphorylated at GSK3 residues S33/37/T41 is more cytoplasmic than nuclear in SW480 cells lacking E-cadherin (Fig. 5). These data may explain why ABC is intrinsically more active in β-catenin signaling assays [13], [14]. Whether phosphorylation of β-catenin at S33/37/T41 promotes cytoplasmic retention or nuclear export is not clear. β-catenin is thought to directly engage the nuclear pore complex due to structural similarities between its central armadillo domain and the HEAT-repeats of the nuclear transport factor, importin-β [32]. While the distribution of β-catenin can be dictated by the availability of nuclear versus cytoplasmic binding partners [33], [34], it has also been shown that first 49 amino acids of β-catenin contain nuclear export information in a *Xenopus* oocyte export assay [35]. β-TrCP (beta-Transducin repeat Containing Protein) is currently the only known factor that recognizes β-catenin phosphorylated at pS33 and pS37. β-TrCP is an F-box protein that is part of the SCF-ubiquitin-ligase complex, where it targets numerous phosphorylated substrates for ubiquitylation and degradation (e.g., β-catenin, IkB, Cdc25A) [36]. As it is the WD40-repeat domain in β-TrCP that recognizes phospho-β-catenin, and WD40 repeats are found in many proteins with different functions (some of which are involved in nuclear export events, [37]), it is possible that β-catenin phosphorylated at S33, S37 and T41 recognizes a WD40 repeat-containing protein that prevents nuclear import or promotes export.

Evidence that cytosolic ABC runs mostly monomeric by sizing chromatography (Fig. 2) may also explain why β-catenin unphosphorylated at S37 and T41 is more active in β-catenin signaling assays [13], [14]. α-catenin is a major stoichiometric binding partner of cytosolic β-catenin [25], and its over-expression can antagonize β-catenin nuclear signaling activity through an incompletely defined mechanism [27], [28], [38]. It is not presently obvious how these N-terminal phosphorylations could promote, or hypo-phosphorylation at these sites could restrict, β-catenin binding to α-catenin. More likely, ABC may be less associated with α-catenin as a consequence of its low cellular concentration.

The prominent nuclear pattern of ABC originally observed in Wnt-activated HEK293T cells [14], together with an absence of junctional staining within intestinal crypt cells [23], raised the possibility that ABC might be used exclusively in nuclear signaling rather than adhesion, as in *C. elegans* [5]. However, the nuclear enrichment of ABC observed in cadherin-negative SW480 cells (Fig. 1) is not apparently due to any reduced capacity to associate with cadherins, since ABC can be readily incorporated into cell contacts in E-cadherin-restored SW480 cells (Fig. 3), as well as other cell types [22], [24], [39]. Thus taken altogether, our data suggest that N-terminally unphospho-β-catenin (ABC) is better at nuclear signaling *not* because it is a cadherin-free form of β-catenin, but rather because of its enhanced nuclear accumulation when present in the cytosol. Although the enhanced nuclear accumulation of ABC is only clearly observed in cells lacking a cadherin (Fig. 3), we reason that this phenomenon also holds true in normal cadherin-expressing cells activated by Wnt, where the cytosolic pool of ABC is below the level of robust immunofluorescence detection. In other words, the analysis of ABC localization in the absence of cadherin provides a condition that permits detection of a nuclear/cytoplasmic distribution phenomenon that is more difficult to detect in the presence of cadherins.

Evidence that ABC can associate with cadherins is not surprising, given that the N-terminal GSK3 regulatory domain lies well outside the binding interface between β-catenin and the cadherin [40]. More curious is why the entire cadherin-bound pool of β-catenin is not unmodified at S37 and T41, given that β-catenin becomes rapidly associated with a cadherin during biosynthesis [41]. However, evidence that monoclonal antibody 8E7 (which recognizes ABC) can detect Wnt-induced changes in the cytosolic pool of β-catenin, without needing to remove the more abundant cadherin-bound β-catenin by fractionation methods [14], implies that β-catenin unmodified at S37 and T41 may not be the major form of β-catenin associated with cadherins. Since β-catenin phosphorylated at S33/37/T41 does not associate with E-cadherin by co-immunoprecipitation and fractionation analyses (Fig. 4), it is formally possible that the β-catenin associated with cadherins may be modified at the S37 and T41 epitope by something other than phosphorylation (e.g., O-glycosylation, [42], [43]; Fig. S4).

Recent studies show that β-catenin phosphorylated at S552 or S675 exhibits enhanced β-catenin signaling activities [15], [16], [17], however, this is not apparently through generating cadherin-free forms of β-catenin, as both phospho-forms can associate with E-cadherin contacts (Fig. 6). In fact, the abundant and distinct localization of these phospho-forms at cell contacts suggests the possibility for phosphorylation-dependent recruitment of factors that mediate junctional maturation and/or signaling.

The spatial uncoupling of β-catenin phosphorylated at T41/45 from that phosphorylated at S33/37/T41 is particularly interesting, because it suggests that phosphorylation at S45 serves a function beyond simply priming β-catenin for subsequent phosphorylations by GSK3. We speculate that the prominent nuclear accumulation of β-catenin phosphorylated at T41/S45 (Fig. 5), together with evidence that it is largely a cadherin-free form of β-catenin (Fig. 4), raises the possibility that phospho-S45 may be a dedicated signaling form of β-catenin. Specifically, phosphorylation by CK1 could both positively and negatively impact β-catenin signaling, such that phosphorylation at S45 promotes transcription, but also primes β-catenin for phosphorylation-dependent degradation by GSK3. Such tight coupling of phospho-activation with degradation could allow for strict temporal control of β-catenin signaling, as observed in other systems (reviewed in [44]). Unfortunately, efforts to tease out a specific contribution of S45 phosphorylation using luciferase reporter assays was confounded by the pleiotropic effects of overexpressing CK1 isoforms. Perhaps generation of an antibody that recognizes β-catenin specifically unphosphorylated at S45, together with current antibodies that recognize phospho-S45 in chromatin immunoprecipitation analysis of β-catenin/TCF target genes would enable the field to answer this question.

## Materials and Methods

### Cell culture and antibodies

SW480 cells were obtained from American Type Culture Collection (ATCC). SW480 mock and E-cadherin transfected cells were described in [22], [45]. An shRNA for α-catenin (GATCCGCCAGTCCAGGTGGTGAATTTTTTCAAGA GAAAAATTCACCAC**/G**CTGGACTGGTTTTTTACG) and a single mutant control sequence (bolded in the previous sequence) was ligated into the pSIREN vector (Invitrogen), transfected into SW480 cells using Lipofectamine (Invitrogen) and stably selected using 2.5 µg/mL puromycin. Antibodies used in this study are as follows: total β-catenin (monoclonal) and α-catenin (BD Biosciences/Transduction labs); total β-catenin (polyclonal) and GAPDH (Santa Cruz); active β-catenin (ABC, clone 8E7 Upstate/Millipore); α-catenin polyclonal (Barry Gumbiner, University of Virginia); Axin (Roel Nusse, Stanford and Zymed); P33/37/41, P41/45, P45, GSK-3β, LRP6, P552, P675 (Cell Signaling); mouse and rabbit IgGs (Chemicon); β-tubulin (Sigma); E-cadherin (HECD-1, Zymed); Alexa Fluor 488 and 568-conjugated goat IgGs, streptavidin-HRP (Horseradish Peroxidase) conjugate (Invitrogen); goat anti-mouse and anti-rabbit IgG-HRP conjugates (Bio-Rad); anti-flag M2 (Sigma).

### Immunofluorescence

Cells were fixed in ice-cold anhydrous methanol and processed using standard procedures. Coverslips were mounted with Aqua Poly/Mount (Polysciences). Images were captured with the Axioplan 2 microscope (Zeiss) equipped with a 63× Plan-Neofluar, NA 1.25 objective, the AxioCam HRm camera, AxioVision4.6 software (Zeiss).

### Affinity precipitation and Western blotting

Cells were lysed in buffer containing 50 mM Tris, pH 7.5, 5 mM EDTA, 150 mM NaCl, 5% glycerol and 1% Triton-X100 with protease inhibitor cocktail (Roche) and 1 mM microcystin (Calbiochem). For cad-GST binding experiment, lysate was incubated with 1 µg of cadherin-GST precoupled to glutathione sepharose (GE Healthcare). For immunoprecipitations, lysates were incubated with indicated antibodies and ImmunoPure Immobilized Protein G or Protein A (Pierce). Precipitated proteins were washed and subjected to SDS-PAGE and Western blot analysis using standard procedures. Densitometry was performed using the CanoScan software (Canon) and analyzed using Image J (National Institutes of Health).

### Cellular fractionation and sucrose equilibrium density gradient centrifugation

Detergent-free lysis was performed as previously described [22] to generate cytosolic and membrane fractions. Cytosolic fractions were TCA precipitated prior to Western blot analysis. Membrane fractions were either resuspended in PBS and analyzed by Western blot, or were subjected to sucrose density gradient centrifugation, performed according to [22].

### [^35^S]-methionine/cysteine metabolic labeling

Steady state metabolic labeling of proteins with [^35^S]-methionine/cysteine was done according to [25]. Briefly, cells were labeled overnight with 1–2 mCi/10-cm dish Redivue PRO-MIX [^35^S] cell labeling mix (Amersham). Cells were lysed in 1% Triton-X100 buffer described above prior to immunoprecipitation, and proteins were separated by SDS-PAGE on Criterion gels (Bio-Rad).

### Gel filtration chromatography

Gel filtration chromatography was performed according to [25]. Briefly, the cytosolic fraction was separated on a Hi Prep 16/60 Sephacryl S-300 sizing column (Amersham Biosciences; High Resolution Code 17-1167-01, 10–1500 kD inclusion range) equilibrated with buffer containing 30 mM Hepes, pH 7.5, and 150 mM KCl and developed at 0.3 ml/min. 1.5-ml fractions were collected and TCA precipitated prior to Western blot analysis.

### Surface biotinylation

Surface biotinylation was performed as described elsewhere [46]. In brief, cells were rinsed in PBS supplemented with 0.1 mM CaCl_2_ and 1 mM MgCl_2_ (PBS^++^), labeled in the dark on ice for 20 minutes with 1 mg/mL EZ-link Sulfo-NHS-LC-biotin (Pierce) in a solution of 10 mM triethanolamine, pH 8, 2 mM CaCl_2_, and 150 mM NaCl. The reaction was quenched with 100 mM glycine in PBS^++^, and cells were rinsed in PBS^++^ prior to lysis with 1% Triton buffer described above. Lysates were immunoprecipitated with antibodies as indicated, and Western blots were performed using streptavidin-HRP (Invitrogen).

### Phospho-peptide mapping

Cytosolic β-catenin was affinity precipitated with 100 µg GST-ICAT from SW480 controls cell lysate (approximately 50 mg total protein) and subjected to SDS-PAGE. Purity of protein preparation was confirmed by Coomassie stain, and the band corresponding to β-catenin was excised and sent to the Taplin Facility for Mass Spectrometry (Harvard). Trypsin digested peptides were identified by LC-MS/MS.

### Phosphatase experiment

Immunoprecipitated complexes were treated with lambda protein phosphatase (New England BioLabs) according to manufacturer's instructions. Samples were also incubated with phosphatase inhibitors: 10mM sodium vanadate (tyrosine, Sigma) and 50 mM sodium fluoride (serine/threonine, Sigma), if indicated. Reaction was performed at 30°C for 30 minutes and quenched with Laemmli sample buffer prior to Western analysis.

## Supporting Information

Figure S1 Recombinant β-catenin runs as a monomer by gel filtration chromatography. Histidine and 6-myc tagged β-catenin (∼2 mg) was purified from baculovirus, diluted in column equilibration buffer and injected onto a sizing column. 1.5-ml fractions were collected and TriChloroacetic Acid (TCA) precipitated prior to Western blot analysis with anti-myc antibodies. Peak fraction (#38) is denoted by an arrow and reveals that the peak corresponding to monomeric β-catenin seen in Figures 2 and 3 is likely due to uncomplexed β-catenin. Note that the purified 6-myc β-catenin sizes larger than monomeric β-catenin from SW480 cytosol due to the presence of histidine and myc epitope tags, which retard β-catenin mobility in SDS-PAGE by ∼20 kDa.
(0.43 MB TIF)

Figure S2 N-terminally phosphorylated β-catenin does not associate with a GST-cadherin cytoplasmic domain by affinity precipitation. A detergent-free cytosolic fraction from SW480 cells was sequentially incubated with GST-cadherin cytoplasmic domain coupled-glutathione sepharose beads. Non-binding lane contains the total unbound fraction precipitated with Trichloroacetic Acid. Note that while a significant fraction of total β-catenin can be affinity precipitated by GST-cadherin, β-catenin phosphorylated at S33/37/T41 does not associate.
(0.38 MB TIF)

Figure S3 Specificity of phospho-β-catenin antibodies in SW480 cells. SW480 cells were transfected with siRNAs against human β-catenin (L-003482-00-0005) ON-TARGETplus SMARTpool siRNA (Thermo Scientific Dharmacon) or non-targeting control sequences (D-001810-10-05) using DharmaFECT reagent (Thermo). After 48 hours, cells were solubilized and subjected to SDS-PAGE immunoblot analysis with the antibodies specified. GAPDH protein levels (control) do not change upon β-catenin silencing (not shown). Note that the phospho-β-catenin antibodies almost exclusively recognize a single band over a 15–200 kDa range, and this band disappears upon β-catenin silencing. The ∼120 kDa band detected with the P33/37/41 antibody is typically much less abundant than phospho-β-catenin [22]. We have previously determined that the ∼160 kDa band detected with the ABC antibody (*) does not account for the nuclear staining in SW480s, although may be an issue in other cell types [47]. Staining patterns observed for all phospho-β-catenin antibodies are diminished by β-catenin silencing by siRNA (not shown) or genetic ablation [22].
(2.33 MB TIF)

Figure S4 Phosphatase treatment of total β-catenin removes N-terminal phosphorylations but does not unmask the ABC epitope. SW480 cells were solubilized in 1% TX-100 lysis buffer and total β-catenin was immunoprecipitated. Reactions were divided and treated with and without lambda phosphatase or phosphatase inhibitors for 30 minutes. Reaction was quenched with sample buffer prior to SDS-PAGE. Phosphatase treatment does not appear to unmask the ABC epitope, but does remove N-terminal phosphates. Note that longer incubation times (up to 18 hours) were still unable to unmask the ABC epitope, despite evidence to the contrary by Hendriksen et al [39].
(0.72 MB TIF)
