# Supplementary figures and images for: β-Catenin Phosphorylated at Serine 45 Is Spatially Uncoupled from β-Catenin Phosphorylated in the GSK3 Domain: Implications for Signaling
Source: PLoS One. 2010 Apr 16;5(4):e10184. doi: 10.1371/journal.pone.0010184 (PMC2855705; doi:10.1371/journal.pone.0010184)

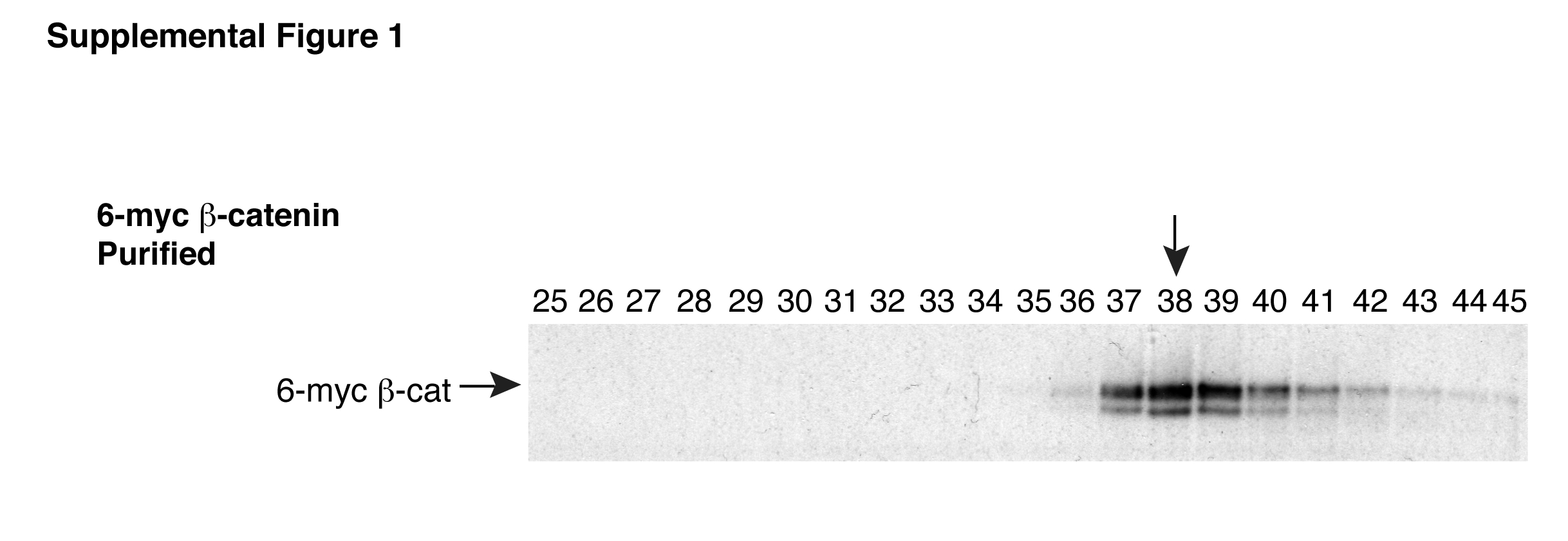

Supplement: Figure S1 — Recombinant β-catenin runs as a monomer by gel filtration chromatography. Histidine and 6-myc tagged β-catenin (∼2 mg) was purified from baculovirus, diluted in column equilibration buffer and injected onto a sizing column. 1.5-ml fractions were collected and TriChloroacetic Acid (TCA) precipitated prior to Western blot analysis with anti-myc antibodies. Peak fraction (#38) is denoted by an arrow and reveals that the peak corresponding to monomeric β-catenin seen in Figures 2 and 3 is likely due to uncomplexed β-catenin. Note that the purified 6-myc β-catenin sizes larger than monomeric β-catenin from SW480 cytosol due to the presence of histidine and myc epitope tags, which retard β-catenin mobility in SDS-PAGE by ∼20 kDa. (0.43 MB TIF) [file pone.0010184.s001.tif]

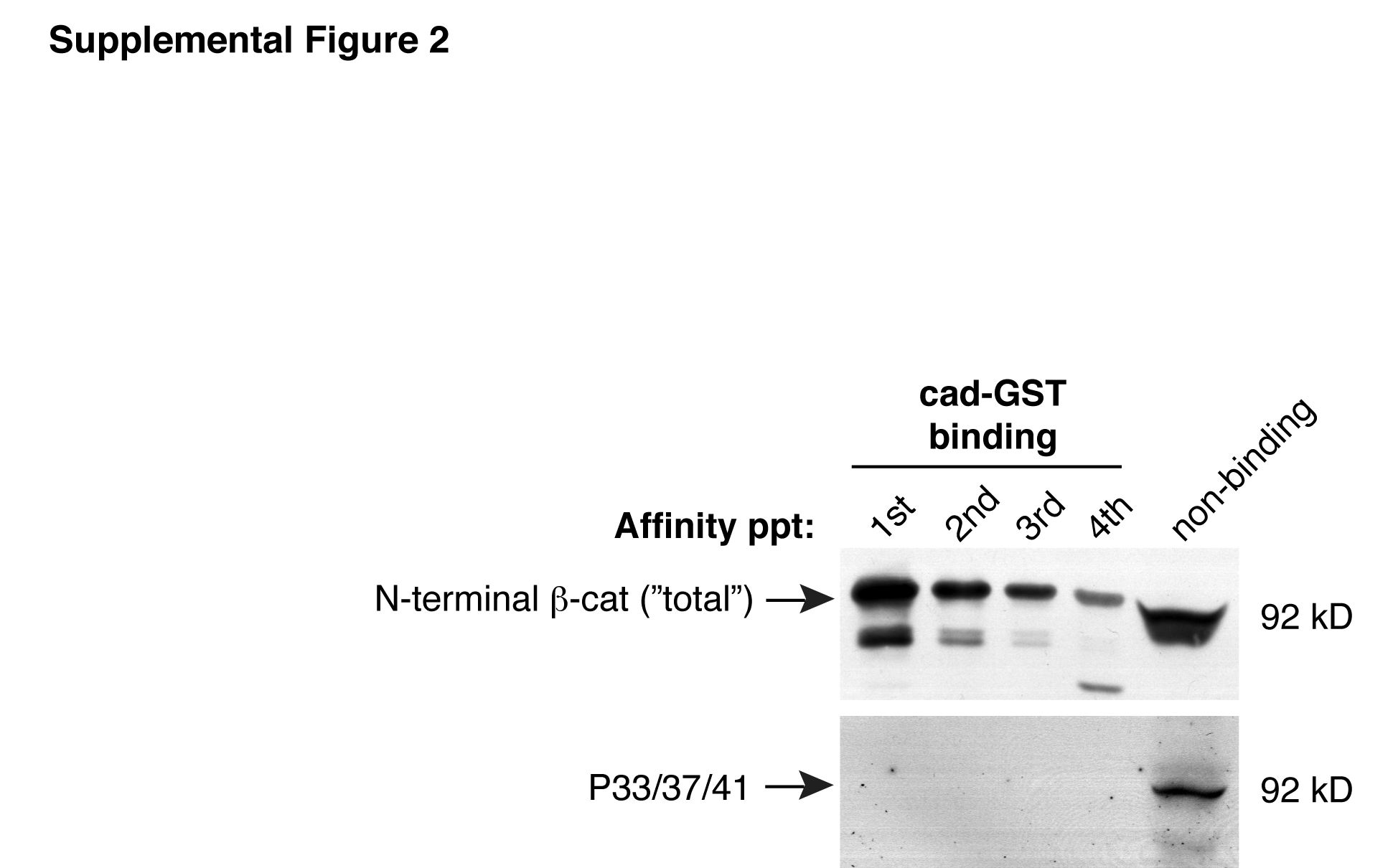

Supplement: Figure S2 — N-terminally phosphorylated β-catenin does not associate with a GST-cadherin cytoplasmic domain by affinity precipitation. A detergent-free cytosolic fraction from SW480 cells was sequentially incubated with GST-cadherin cytoplasmic domain coupled-glutathione sepharose beads. Non-binding lane contains the total unbound fraction precipitated with Trichloroacetic Acid. Note that while a significant fraction of total β-catenin can be affinity precipitated by GST-cadherin, β-catenin phosphorylated at S33/37/T41 does not associate. (0.38 MB TIF) [file pone.0010184.s002.tif]

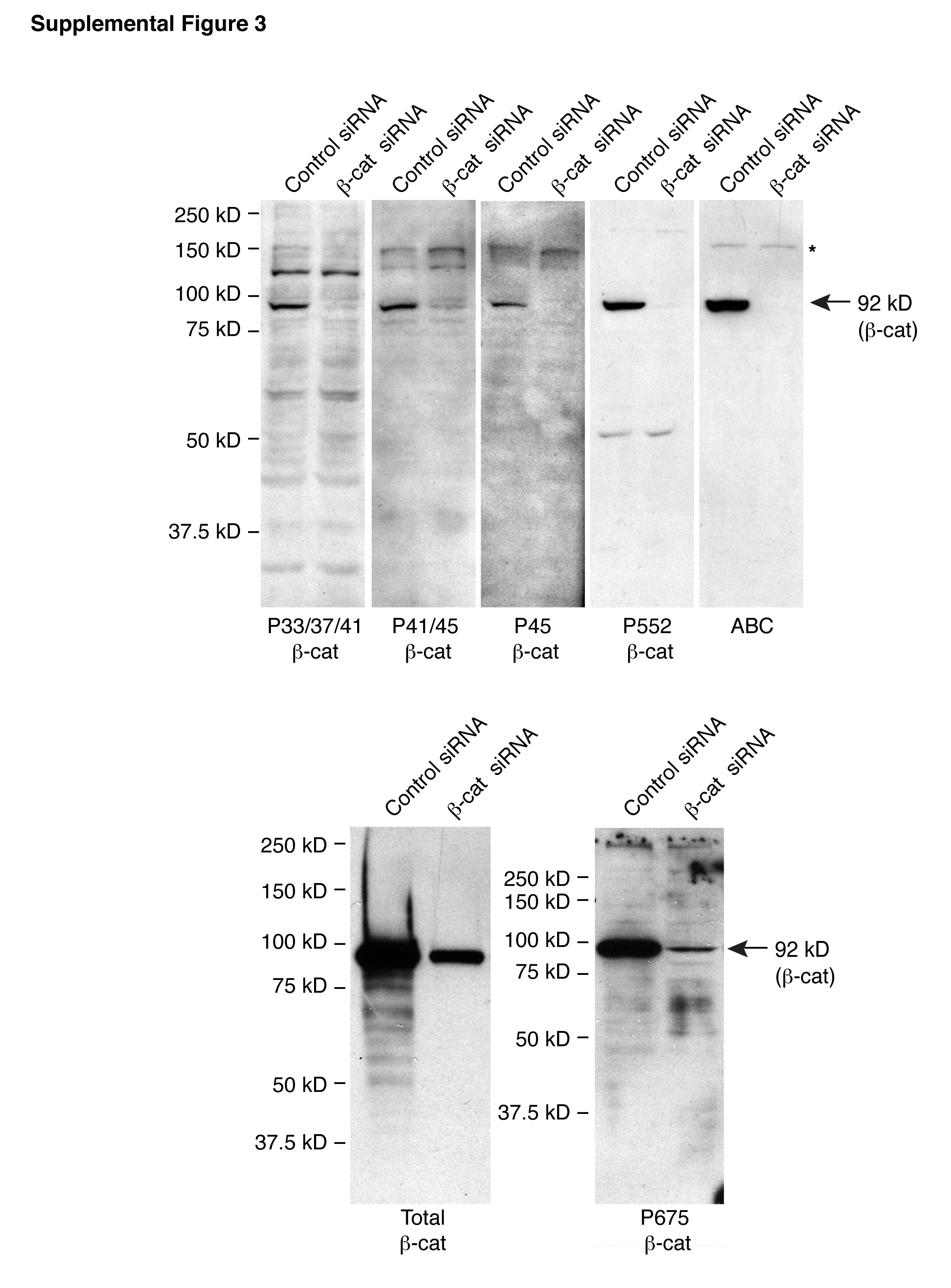

Supplement: Figure S3 — Specificity of phospho-β-catenin antibodies in SW480 cells. SW480 cells were transfected with siRNAs against human β-catenin (L-003482-00-0005) ON-TARGETplus SMARTpool siRNA (Thermo Scientific Dharmacon) or non-targeting control sequences (D-001810-10-05) using DharmaFECT reagent (Thermo). After 48 hours, cells were solubilized and subjected to SDS-PAGE immunoblot analysis with the antibodies specified. GAPDH protein levels (control) do not change upon β-catenin silencing (not shown). Note that the phospho-β-catenin antibodies almost exclusively recognize a single band over a 15–200 kDa range, and this band disappears upon β-catenin silencing. The ∼120 kDa band detected with the P33/37/41 antibody is typically much less abundant than phospho-β-catenin [22]. We have previously determined that the ∼160 kDa band detected with the ABC antibody (*) does not account for the nuclear staining in SW480s, although may be an issue in other cell types [47]. Staining patterns observed for all phospho-β-catenin antibodies are diminished by β-catenin silencing by siRNA (not shown) or genetic ablation [22]. (2.33 MB TIF) [file pone.0010184.s003.tif]

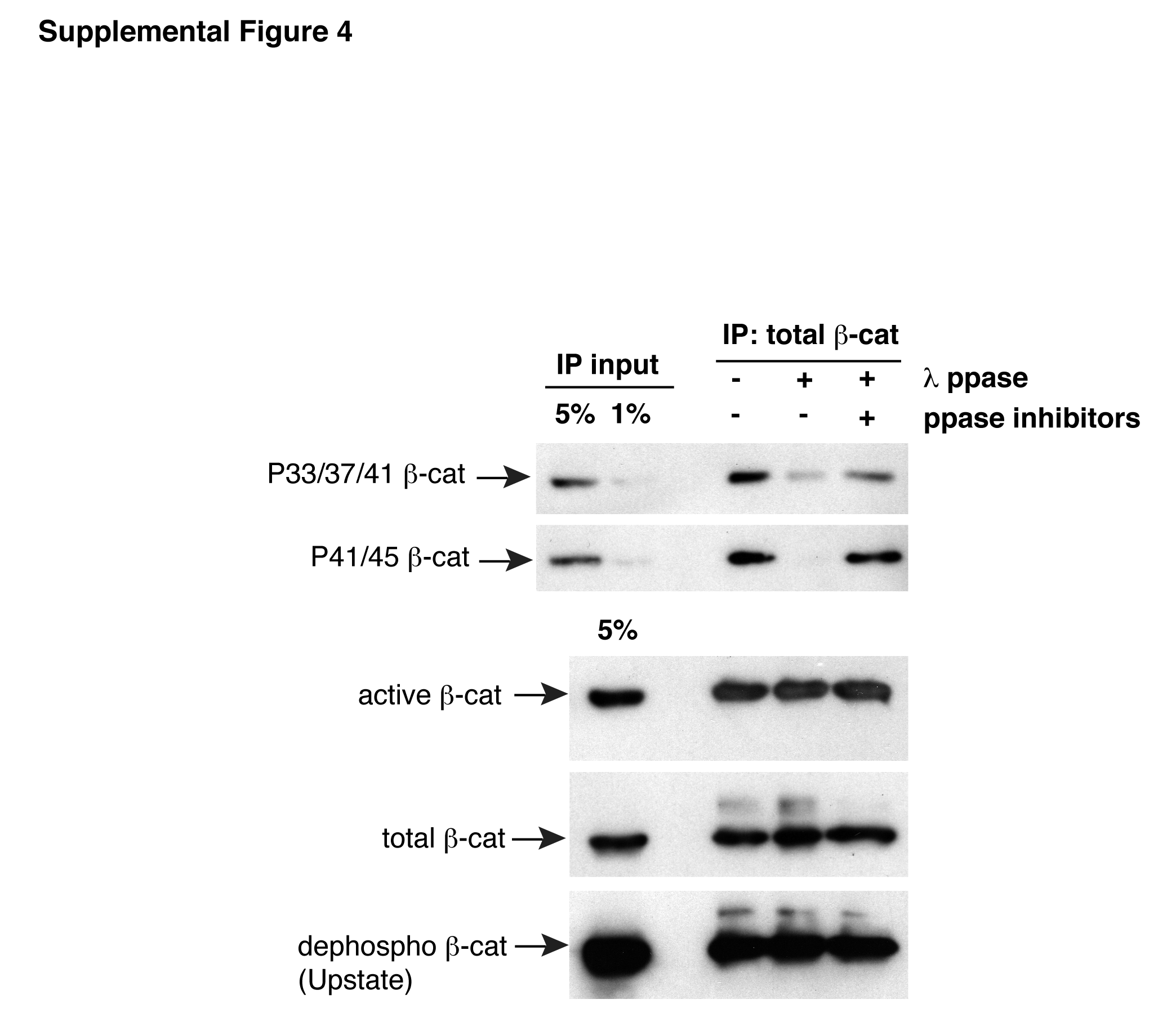

Supplement: Figure S4 — Phosphatase treatment of total β-catenin removes N-terminal phosphorylations but does not unmask the ABC epitope. SW480 cells were solubilized in 1% TX-100 lysis buffer and total β-catenin was immunoprecipitated. Reactions were divided and treated with and without lambda phosphatase or phosphatase inhibitors for 30 minutes. Reaction was quenched with sample buffer prior to SDS-PAGE. Phosphatase treatment does not appear to unmask the ABC epitope, but does remove N-terminal phosphates. Note that longer incubation times (up to 18 hours) were still unable to unmask the ABC epitope, despite evidence to the contrary by Hendriksen et al [39]. (0.72 MB TIF) [file pone.0010184.s004.tif]
